# Not a “they” but a “we”: The microbiome helps promote our well-being

**DOI:** 10.1016/j.jbc.2021.101511

**Published:** 2021-12-18

**Authors:** Stephen W. Ragsdale

**Affiliations:** Department of Biological Chemistry, University of Michigan Medical School, Ann Arbor, Michigan, USA

**Keywords:** microbiome, methyltransferase, demethylase, vitamin B_12_, trimethylamine-N-oxide, trimethylamine, γ-butyrobetaine, Eubacterium limosum, short chain fatty acid, Wood–Ljungdahl pathway, methyl-H_4_folate, methyltetrahydrofolate, TMA, trimethylamine, TMAO, trimethylamine-*N*-oxide

## Abstract

Anaerobic microbes in the human gut produce beneficial and harmful compounds, as well as neutral compounds like trimethylamine, which undergoes microbial metabolism or host-catalyzed transformation into proatherogenic trimethylamine-*N*-oxide. Ellenbogen *et al.* identified a microbiome-associated demethylase that short-circuits the production of trimethylamine-*N*-oxide from the metabolite γ-butyrobetaine and instead produces methyltetrahydrofolate, a key intermediate in the microbial production of beneficial small-chain fatty acids. This article highlights an example of how the microbiome is integrally involved in producing metabolites that support our health and in preventing the formation of compounds that promote disease.

The human microbiome is a rich network of bacteria, archaea, fungi, phages, and viruses living mostly in the intestines. The composition of the microbiome can determine our own well-being (1), prompting an explosion of research efforts focused on identifying the constituent organisms and defining their metabolic pathways, metabolites, and the association of individual genes with health or disease. For example, the amino acids lysine and methionine in our diet are metabolized into l-carnitine, which is then converted into γ-butyrobetaine and trimethylamine (TMA). TMA is absorbed into the bloodstream and then undergoes enzymatic oxidation in the liver to form trimethylamine-*N*-oxide (TMAO) (Fig. 1). TMAO levels are correlated with cardiovascular and kidney diseases as well as colorectal and liver cancer (2). Thus, all three of these metabolites (γ-butyrobetaine, TMA, and TMAO) can be associated with the disease. In a recent publication in the *Journal of Biological Chemistry*, Ellenbogen *et al.* (3) have uncovered a novel activity of the enzyme MtyB from the gut microbe *Eubacterium limosum* that potentially interrupts the formation of TMAO by removing a methyl group from the abundant cellular metabolite γ-butyrobetaine to generate methyltetrahydrofolate (methyl-H_4_folate) (3), a precursor of the short-chain fatty acid, acetic acid.

**Figure 1:**
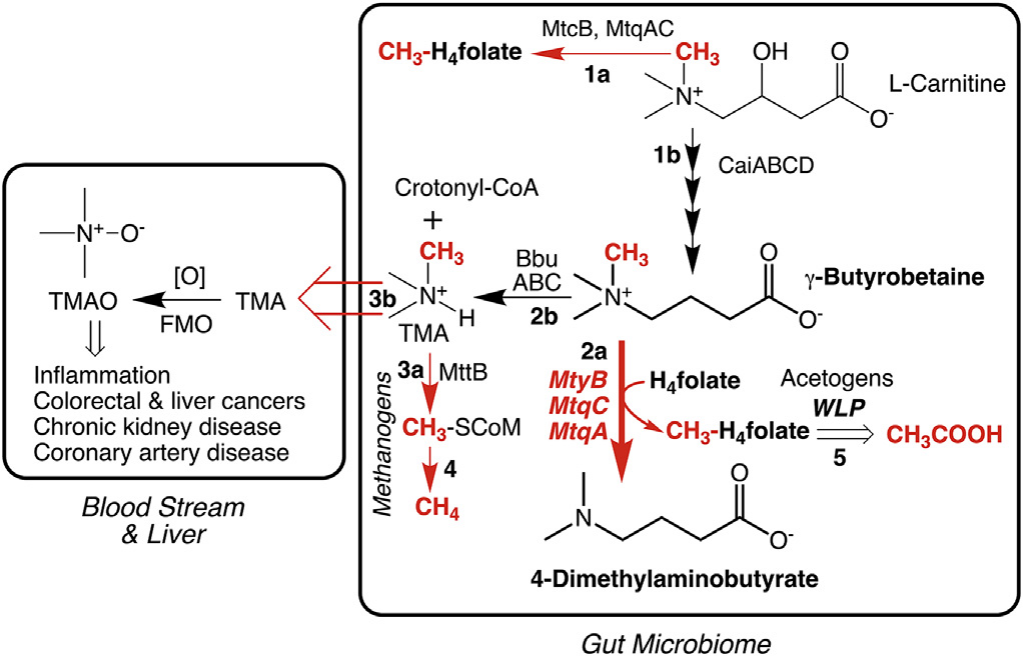
**Three microbiome reactions protect against host formation of disease-promoting TMAO.** The MtcB/MtqAC methyltransferase (Rxn 1a) (5) produces CH_3_-H_4_folate from the metabolite carnitine, whereas CaiABCD (Rxn 1b) (9) generates γ-butyrobetaine. Similarly, γ-butyrobetaine undergoes metabolism by either the MtyB/MtqAC methyltransferase (Rxn 2a) described by Ellenbogen *et al.* (3) or BbuABC (Rxn 2b) (7) for generation of CH_3_-H_4_folate or TMA. Entry of TMA into the blood stream, where a host monooxygenase catalyzes its oxidation to TMAO (Rxn 3b), is counterbalanced by microbial MttB-catalyzed conversion to CH_3_-CoM (Rxn 3a) (10). Further microbial metabolism of CH_3_-CoM or CH_3_-H_4_folate leads to methane (Rxn 4) or acetic acid (Rxn 5), a beneficial SCFA. H_4_folate, tetrahydrofolate; Mtc methyltransferases for carnitine; Mtt, methyltransferases for trimethylamine; Mty, methyltransferases for γ-butyrobetaine; SCFA, short-chain fatty acid; TMA, trimethylamine; TMAO, trimethylamine-*N*-oxide; WLP, Wood–Ljungdahl pathway.

*E. limosum* is typically associated with good health and longevity, perhaps partly because of its production of short-chain fatty acids, which are known to serve beneficial functions in human health, including providing fuel for growth of colonocytes (4). As suggested by the authors (3), it seems likely that that the butyrobetaine demethylase system is partly responsible for the microbe's beneficial health effects by lowering the levels of proatherogenic TMA and TMAO in the bloodstream. The reason for stating that this system only “partly” contributes to the beneficial effects of *E. limosum* is that this new finding from the Krzycki laboratory comes on the heels of their discovery of a carnitine demethylase system (MtqA, MtcB, and MtqC) (5) that also produces acetate. In the research highlighted here, Ellenbogen *et al.* (3) described a three-protein complex including two of the same proteins (MtqA and MtqC) that associate with MtyB to remove a methyl group from γ-butyrobetaine (Fig. 1) producing methyl-H_4_folate, which is subsequently converted to acetic acid. Furthermore, they performed proteomic analysis and found that *E. limosum* expresses at least 40 other methyltransferases, related to MtyB, as well as to MttB, distinguished as the founding member of a 10,000-protein family and of being the protein in which the 22nd amino acid (pyrrolysine) was discovered (6).

Ellenbogen *et al.* cultivated *E. limosum* on medium containing several different quaternary amines, including γ-butyrobetaine. They then compared the expression patterns of cells grown on butyrobetaine *versus* the lactate control in a proteomic study, which revealed high levels of MtyB, MtqC, and MtqA expression. This triumvirate is a familiar pattern in vitamin B_12_-dependent methyltransferase systems. As shown in Figure 3 of the highlighted article, these arrangements contain one protein (B) that binds and demethylates a specific substrate, another protein (C) that binds the methyl acceptor B_12_, and finally a third component (A), which catalyzes the methyl group transfer from methylated vitamin B_12_ to a cofactor. In bacteria and humans, this cofactor is H_4_folate, whereas in methanogenic archaea, it is coenzyme M (methyl transfer to coenzyme M forms methyl-coenzyme M, the direct precursor of methane). Through *in vitro* kinetic analyses with purified enzymes, Krzycki *et al.* demonstrated that this three-component system catalyzes the methylation of H_4_folate by butyrobetaine to generate methyl-H_4_folate.

A potential conundrum arose when Ellenbogen *et al.* discovered that this newly identified B-component, MtyB, shares high sequence similarity to the carnitine methyltransferase (MtcB) that also binds MtqA and MtqC (5). Surprisingly, MtyB even exhibits a slightly higher specificity for carnitine over γ-butyrobetaine. This suggested that MtyB might be better classified as a carnitine methyltransferase; however, they demonstrated that MtyB neither was even detectable in cells grown on medium with carnitine nor was MtcB detectable in cells grown on medium with γ-butyrobetaine. Finally, they showed that expression of MtyB required the coexpression of MtqC and that MtyB and MtqC formed a complex that could be purified. These experiments clearly substantiated their conclusion that MtyB, MtqC, and MtqA act as a γ-butyrobetaine:H_4_folate methyltransferase system.

As shown in Figure 1, consumption of γ-butyrobetaine by MtyB and of carnitine by MtcB by *E. limosum* and other microorganisms in the microbiome might provide a natural competitive mechanism to lower the production of proatherogenic metabolites TMA and TMAO. TMA is produced predominantly by the recently discovered anaerobic *bbu* pathway (BbuABCDEF) (7), with TMAO generated by a host monooxygenase. On the other hand, the methyl transfer reactions produce methyl-H_4_folate, which couples directly to the Wood–Ljungdahl pathway for the synthesis of acetyl-CoA (8), which in turn is linked to biomass generation and energy conservation for the bacteria as well as the synthesis of beneficial short-chain fatty acids. Similarly, demethylation of TMA by MttB in methanogenic microbes to produce methane would also divert this substrate from TMAO production.

These methyltransferase pathways are not unique to the guts of humans or even mammals. They are found along with the acetogenic and methanogenic pathways in many diverse anaerobic environments, including the rumen of cows, freshwater and marine sediments, and within soil. The findings of Ellenbogen *et al.* also suggest the possibility that microbes catalyzing demethylation reactions coevolved with their hosts and were selected for to reduce the levels of TMA and TMAO. In the future, it will be important to use animal models and cell cultures to determine how *mttB* silencing and overexpression affects development and progression of TMAO-linked diseases. Furthermore, elucidation of the functions of other MttB-related methyltransferases, including the 40 others currently identified in *E. limosum*, should lead to a greater understanding of how these reactions and how the microbiome benefits human health.

This demethylation system outlined by Ellenbogen *et al.* is another example of how the microbiome serves as a functional organ within the large intestine. The microbiome is part of us, not a “they” but a “we,” integrally involved in controlling the basic chemicals of life, producing and channeling metabolites into pathways that support our health, and preventing us from generating compounds that initiate myriad diseases like cancer and heart disease.

## Conflict of interest

The authors declare that they have no conflicts of interest with the contents of this article.
